# Methodological Considerations in Assessing Mental Health Burden Among Caregivers of Special Needs Children

**DOI:** 10.5041/RMMJ.10570

**Published:** 2026-01-28

**Authors:** Harikrishnan Balakrishna

**Affiliations:** Medical Officer/Assistant Surgeon, Department of Health Services, Government of Kerala, Family Health Centre (FHC) at Kilikolloor, Kollam, Kerala, India

**Keywords:** Caregiver burden, control groups, India, research methodology, socioeconomic confounding, special needs children

## To the Editor

I read with interest the paper by Priya et al. and appreciate their study examining the mental health status among parental caregivers of children with special needs in Puducherry.^1^ The authors addressed an important and understudied population in the Indian caregiver context. Their work made several noteworthy contributions that deserve recognition.

Their comprehensive approach employing four validated instruments—the Zarit Burden Interview, Depression Anxiety Stress Scale (DASS-21), Pittsburgh Sleep Quality Index, and World Health Organization Quality of Life-BREF (WHOQOL-BREF), while comparable Indian studies utilize only one or two assessment tools—signifies excellent methodological rigor. Priya et al. employed WHO-recommended translation protocols, ensuring cultural and linguistic validity. The inclusion of multiple diagnostic categories reflects real-world special education settings and enhances its generalizability. The focus on Puducherry addresses a notable geographical gap, as most Indian caregiver research concentrates on metropolitan centers, and the successful recruitment of 66 caregivers and systematic data collection procedures all reflect a commendable research execution while under resource constraints.

The authors acknowledged several limitations, including the absence of control groups and a single-center design. I respectfully offer some additional considerations for exploration, as these may strengthen future research in this important area.

Priya et al.’s study design presents an opportunity to further clarify the specific contribution of caregiving demands to observed mental health outcomes. The absence of a control group, as acknowledged in their limitations section, merits deeper consideration for interpreting the reported findings. They reported that 89.4% of participants experienced depression and anxiety, 93.9% had poor sleep quality, and quality of life scores ranged from 39 to 43 across domains. Without comparison data from caregivers of typically developing children, particularly those matched on socioeconomic characteristics, these rates cannot establish whether caregiving for special needs children imposes a burden beyond baseline levels in similar populations. Recent trends in international studies are to employ case-controlled designs to establish caregiving-specific effects. Reilly et al. demonstrated that 72% of mothers of children with epilepsy scored in the at-risk range on DASS-21 subscale, with significantly higher rates than matched controls with neurodisability on depression (55% versus 27%, *P*=0.005) and stress (55% versus 33%, *P*=0.03).^2^ While identifying appropriately matched control groups in resource-limited settings presents significant challenges, comparison data would strengthen conclusions about caregiving-specific effects and inform whether targeted caregiver interventions are required or whether broader community mental health support would be more appropriate.

This methodological consideration becomes particularly important given the socioeconomic composition of the sample. Close to half of the participants (47%) earned below India’s urban poverty line (≤INR 15,000/month), with an additional 36.4% declining to report income data. Without statistical control for socioeconomic status as an independent predictor, it remains unclear to what extent poverty and caregiving demands independently and interactively contribute to the observed mental health burden. This distinction carries important implications because poverty independently predicts depression, anxiety, poor sleep quality, and reduced quality of life in general populations.^3^,^4^ The reported quality of life scores of 39–43 fall within ranges that may reflect socioeconomic disadvantage. Understanding whether observed mental health difficulties arise primarily from economic hardship, caregiving-specific factors, or their interaction would inform intervention design and resource allocation. If the burden relates substantially to socioeconomic factors, interventions might appropriately emphasize economic support, employment assistance, and poverty alleviation strategies alongside caregiver-specific services. Poverty alleviation interventions, such as the disability support and social security schemes employed by certain states like Kerala, might effectively reduce caregiver distress independent of caregiver-specific interventions. However, without research designs that control for socioeconomic status, neither the contribution of poverty to burden nor the potential buffering effects of social welfare programs can be adequately evaluated. Conversely, establishing a caregiving-specific burden would justify prioritizing caregiver counseling and respite care. Multivariate factor analysis controlling for household income, parental education level, employment status, and household composition would help disentangle these influences. The potential interactive effects between poverty and caregiving require investigation through designs capable of examining these relationships. This highlights an important gap common in Indian caregiver research.^1^,^5^,^6^

Additionally, the study design would benefit from incorporating child characteristic data. No information was collected regarding disability severity, behavioral problems, functional independence, or care intensity requirements. While not discussed in the article, and though reasons may have included institutional review board restrictions, instrument availability, or consent requirements, this represents an opportunity for future enhancement. A systematic review identified child behavioral difficulties and disability severity as primary predictors of caregiver burden across neurodevelopmental conditions.^7^ Some Indian studies, including work by Patel et al. on families of children and adolescents with autism spectrum disorder, have successfully incorporated measures of child symptom severity or functional status.^5^ Such data would enable identification of which caregivers face the greatest risk, what child-level factors might serve as intervention targets, and whether burden varies more by disability severity within diagnoses than between diagnostic categories. This information would support efficient allocation of limited support services, identification of modifiable risk factors for preventive intervention, and understanding of relationships between care demands and caregiver outcomes.

These methodological considerations, when viewed together, highlight opportunities to strengthen clinical utility. The study documents high rates of mental health symptoms among caregivers, which is an important descriptive finding, but the design limits the ability to determine: (1) the extent to which observed distress is caregiving-specific; (2) whether distress arises mainly from caregiving demands, poverty, or their interaction; and (3) which caregiver or child characteristics predict the greatest risk and might inform a targeted intervention. Addressing these questions would provide clinicians and policymakers with evidence-based guidance for resource allocation and identification of families who might benefit most from targeted support.

The challenges of conducting caregiver research in such resource-limited settings are great, and we genuinely appreciate Priya et al.’s important contribution to this understudied population. The successful recruitment and comprehensive assessment of 66 caregivers represent valuable foundational work. Future studies might strengthen the evidence base by including: (1) control groups of caregivers of typically developing children, ideally matched on key socioeconomic indicators; (2) comprehensive measurement and statistical control of socioeconomic factors; (3) assessment of child behavioral problems, disability severity, and functional independence using validated instruments; and (4) multivariate analyses to identify independent predictors of caregiver outcomes and explore potential interaction effects. These enhancements would help establish whether caregiving for special needs children imposes mental health burdens beyond socioeconomic disadvantage alone, and under what circumstances, and identify which families might benefit most from support services. This study has made a significant contribution by initiating an important conversation about caregiver mental health in Indian special education contexts and demonstrated the feasibility of comprehensive assessment in this population. Building on this foundation with continued methodological development will help the collective understanding and ultimately improve support for families caring for children with special needs.

## Abbreviations

DASS-21: Depression Anxiety Stress Scale
WHOQOL-BREF: World Health Organization Quality of Life-BREF
